# The Fibrinogen-to-Albumin Ratio in Endometriosis: A Step Toward Personalized Non-Invasive Diagnostics

**DOI:** 10.3390/jpm16010020

**Published:** 2026-01-04

**Authors:** Lejla Samson, Theresa Mally, Chiara Paternostro, Alfie Bill, Lorenz Kuessel, Christine Bekos

**Affiliations:** 1 Department of Obstetrics and Gynecology, Medical University of Vienna, 1090 Vienna, Austria; n11810524@students.meduniwien.ac.at (T.M.); lorenz.kuessel@meduniwien.ac.at (L.K.); christine.bekos@meduniwien.ac.at (C.B.); 2 Department of Medical Sciences, Hull York Medical School, York University, York YO10 5DD, UK; hyab61@hyms.ac.uk; 3 Department of Obstetrics and Gynecology, Norfolk and Norwich University Hospital Norwich, Norfolk NR4 7UY, UK

**Keywords:** endometriosis, fibrinogen-to-albumin ratio, systemic inflammation, diagnostic biomarker, adenomyosis, deep infiltrating endometriosis

## Abstract

**Background/Objectives:** Endometriosis is a chronic inflammatory disease affecting up to 10–15% of women of reproductive age and is frequently associated with pelvic pain and infertility. Non-invasive biomarkers remain insufficient for accurate diagnosis, often necessitating laparoscopic confirmation. The fibrinogen-to-albumin ratio (FAR), a composite marker of systemic inflammation, has been proposed in both oncological and cardiovascular disease but has not yet been evaluated in endometriosis. **Methods:** We conducted a retrospective monocentric study including 390 women who underwent laparoscopy between January 2015 and December 2021 at the Medical University of Vienna. Of these, 218 had histologically confirmed endometriosis and 172 had benign ovarian cysts. Preoperative laboratory data was collected, and FAR was calculated. Group comparisons were performed using the Mann–Whitney U test. ANOVA was used to compare FAR across revised American Society for Reproductive Medicine (rASRM) stages, and Spearman’s rank correlation assessed associations with disease severity. Subgroup analyses were performed for adenomyosis and deep infiltrating endometriosis (DIE). **Results:** FAR was significantly higher in women with endometriosis than in controls (median 0.0679, IQR 0.0588–0.0778 vs. 0.0641, IQR 0.0559–0.716; *p* = 0.0035). Across rASRM stages I–IV, FAR values were comparable (means 0.0691–0.0709) and did not differ significantly (*p* = 0.822, ANOVA). Spearman’s correlation confirmed no significant association with disease stage (ρ = 0.085, *p* = 0.24). In exploratory analyses, women with adenomyosis (*n* = 35) showed a non-significant trend toward a higher median FAR compared to those without adenomyosis (0.0707 vs. 0.0669; *p* = 0.073, one-sided). No difference in FAR was observed between women with deep infiltrating endometriosis (DIE; *n* = 144) and those without (0.0680 vs. 0.0672; *p* = 0.389, one-sided). **Conclusions:** Although FAR alone cannot replace surgical confirmation, the difference observed between the groups may reflect the systemic inflammatory aspect of endometriosis and should be investigated further in future studies. Given its accessibility and cost-effectiveness, FAR may support the development of non-invasive, personalized diagnostic approaches when combined with other clinical and molecular markers.

## 1. Introduction

Endometriosis is a chronic, estrogen-dependent inflammatory disease defined by the growth of endometrial-like tissue outside the uterine cavity [1,2]. It affects an estimated 10–15% of women of reproductive age, and up to 50% of women with infertility, making it one of the most common gynaecologic disorders [3,4]. The condition is associated with pelvic pain, dysmenorrhea, dyspareunia, and subfertility, and can profoundly impair quality of life [4,5].

Although Sampson’s classic theory of retrograde menstruation continues to be the most widely cited explanation for the pathogenesis of endometriosis, it does not fully account for the diversity of clinical presentations [6]. Alternative or complementary mechanisms—including coelomic metaplasia, lymphatic or hematogenous dissemination, stem cell involvement, and impaired immune surveillance—have been proposed [7,8,9]. A central feature across these theories is chronic inflammation, which supports lesion establishment and persistence through angiogenesis, immune cell recruitment, and oxidative stress [10,11,12,13,14].

Increasing evidence supports the view of endometriosis as a chronic inflammatory and immune-mediated disease, rather than a purely gynecologic condition [15]. Ectopic endometrial-like tissue provokes aberrant immune responses, leading to a self-sustaining cycle of local inflammation, impaired immune clearance, and lesion persistence [16]. Macrophages, neutrophils, natural killer (NK) cells, and T lymphocytes are all implicated in this dysregulated immune response [17]. In particular, peritoneal macrophages show increased numbers but reduced phagocytic capacity and altered receptor expression, including downregulation of CD36 and upregulation of CD47, facilitating ectopic cell survival [18,19].

Multiple studies have shown that women with endometriosis have elevated levels of pro-inflammatory cytokines in their peritoneal fluid. These include interleukin (IL)-6, tumor necrosis factor-alpha (TNF-α), and sometimes IL-1β and IL-8, which are significantly increased compared to women without endometriosis [20,21]. These cytokines create a local environment that promotes blood vessel formation, tissue growth, and inflammation, all of which support the development and persistence of endometriotic lesions [15]. The inflammatory environment also contributes to symptoms such as pelvic pain and infertility [22].

At the same time, there is evidence that certain immune cells responsible for removing ectopic tissue are less active. For example, natural killer (NK) cells found in the peritoneal fluid of women with endometriosis show reduced cytotoxic activity, partly due to high levels of IL-6, which suppress important killing mechanisms like perforin and granzyme B [23,24]. These changes suggest that both increased inflammation and reduced immune clearance play a role in the disease process.

Deep infiltrating endometriosis (DIE) appears to exhibit a more intense inflammatory profile compared to superficial peritoneal disease, including increased expression of nerve growth factor (NGF), and enhanced fibrosis and neuroangiogenesis [25,26]. These factors may underlie the disproportionate pain symptoms frequently observed in DIE.

Systemically, endometriosis has been associated with altered levels of acute-phase proteins such as fibrinogen and albumin, indicative of broader inflammatory and coagulation-related changes [27]. Elevated fibrinogen and increased thrombin activity, often driven by capillary leakage and hepatic response, have been reported in affected women [10,28]. These systemic alterations form the basis for interest in composite biomarkers such as the fibrinogen-to-albumin ratio (FAR), which has been studied in other chronic inflammatory and autoimmune conditions [29].

Although these mechanisms are increasingly well-described, key gaps remain. The reasons why only some individuals with retrograde menstruation develop lesions are not fully understood [30]. Moreover, the interplay between systemic inflammation, lesion-specific immune responses, and clinical phenotypes, such as DIE or ovarian endometriosis, requires further elucidation [31,32]. Improved characterization of these pathways could inform novel diagnostic and therapeutic strategies.

Diagnosis remains a significant challenge. The current gold standard is laparoscopic visualization followed by histological confirmation, a procedure that is invasive, costly, and often delayed by years after symptom onset [33]. Accordingly, many studies have tried to identify non-invasive biomarkers—from simple serologic markers to complex multimarker panels—to enable earlier and easier diagnosis.

The most widely studied biomarker is CA-125. A 2016 meta-analysis including over 2900 women found that using a threshold of CA-125 ≥ 30 U/mL yields a pooled sensitivity of 52% (95% CI 38–66%) and specificity of 93% (95% CI 89–95%) for endometriosis; sensitivity rose to about 63% in moderate/severe disease vs. only about 24% in minimal disease [34]. Many women with mild endometriosis have normal CA-125 levels and CA-125 may be raised in many benign or physiological conditions (e.g., menstruation, benign cysts), limiting its usefulness for early diagnosis or screening. More recent evaluations continue to reach the same conclusion: although CA-125 may help in cases with advanced or extensive disease, no non-invasive marker, including CA-125, has sufficient diagnostic accuracy across different stages and clinical phenotypes to replace surgical diagnosis [35].

Other systemic markers of inflammation and immune activity have been assessed. For instance, the Neutrophil-to-Lymphocyte Ratio (NLR) has been proposed as a surrogate of systemic inflammation. In a 2024 study, NLR demonstrated a moderate diagnostic performance for pelvic-pain-associated endometriosis: at a cutoff of 1.85, sensitivity was 59% and specificity 71% (AUC 0.63) [36]. Similarly, platelet-to-lymphocyte ratio (PLR) and other hematologic indices have been tested. However, their standalone performance tends to be weak and often insufficient to rule in/out disease reliably, especially considering that many inflammatory markers fluctuate with menstrual cycle, co-morbidities, or physiological changes [37,38].

To overcome these limitations, composite or multi-parameter biomarker panels have been proposed. For example, combining CA-125 with hematologic ratios (e.g., PLR) yielded better diagnostic performance in some studies: in one analysis of ovarian endometriosis, a combined marker (CA-125/PLR) achieved 83.4% sensitivity and 95.8% specificity (AUC ~0.91), outperforming either marker alone [39]. Other novel combinations include serum proteins, cytokines, or even miRNA panels. For example, a 2023 study combined four miRNAs with CA-125 and reported a sensitivity of 81.8% and a specificity of 92.6% with an AUC of 0.94. for diagnosing endometriosis [40]. Nevertheless, while these panels are promising, they often lack external validation, are derived from small or selected cohorts, or show declining performance in early-stage disease, meaning they are not yet ready for widespread clinical use. A 2023 comprehensive review concluded that, as of now, no non-invasive biomarker or panel achieves both high sensitivity and specificity across all disease stages and phenotypes [38].

Further complicating biomarker development is the heterogeneity of endometriosis: different phenotypes (superficial, ovarian endometrioma, deep infiltrating endometriosis), variable lesion burden, and influence of menstrual cycle, hormonal treatment or comorbidities can all affect marker levels [41,42,43]. Therefore, a “one-size-fits-all” biomarker is unlikely. Instead, a validated, possibly multimarker panel, ideally adjusted for phenotype, stage, and individual patient factors, seems more realistic [13,44].

Given these limitations and unmet needs, exploring alternative markers is justified. The Fibrinogen-to-Albumin Ratio (FAR) represents one such candidate, potentially reflecting systemic inflammatory and coagulation pathways. The fibrinogen-to-albumin ratio (FAR) is an emerging marker of systemic inflammation [45,46,47].

During inflammation, the body activates a systemic response known as the acute-phase reaction, which leads to changes in liver protein synthesis. In this process, the production of some plasma proteins increases (called positive acute-phase proteins), while the synthesis of others is reduced (negative acute-phase proteins) [48].

Fibrinogen is one of the main positive acute-phase proteins. Its production in the liver is strongly stimulated by pro-inflammatory cytokines, especially interleukin-6 (IL-6), but also interleukin-1β and tumor necrosis factor-alpha (TNF-α). These cytokines activate intracellular pathways like STAT3, which increase the transcription of fibrinogen genes in hepatocytes [49]. As a result, fibrinogen levels in the blood rise significantly during inflammation. This is not only important for the coagulation system but also reflects a more general pro-inflammatory and pro-thrombotic state in the body [50].

On the other hand, albumin behaves as a negative acute-phase protein. Inflammatory signals reduce its synthesis in the liver, because the metabolic resources are shifted toward the production of positive acute-phase proteins like fibrinogen and C-reactive protein [51,52]. In addition, inflammation increases the permeability of blood vessels, which allows albumin to leak from the bloodstream into the surrounding tissues. This effect leads to a further reduction in measurable albumin levels in the circulation [52]. Albumin may also be broken down more quickly during inflammation, which contributes to its decrease [53].

Altogether, the opposite behavior of fibrinogen and albumin during inflammation reflects the body’s adaptive response to injury or chronic immune activation [54].

Combining these two parameters yields FAR, which has been associated with disease activity and prognosis in malignancies, cardiovascular disease, and autoimmune conditions [45,46,54]. Whether FAR could reflect the inflammatory milieu of endometriosis has not yet been systematically studied.

This study was designed to investigate the diagnostic potential of FAR in women with endometriosis. Specifically, we sought to determine whether FAR differs between patients with endometriosis and those with benign ovarian cysts, whether it correlates with disease severity according to the revised American Society for Reproductive Medicine (rASRM) classification, and whether it is associated with subtypes such as adenomyosis and deep infiltrating endometriosis.

## 2. Materials and Methods

**Figure A1:**
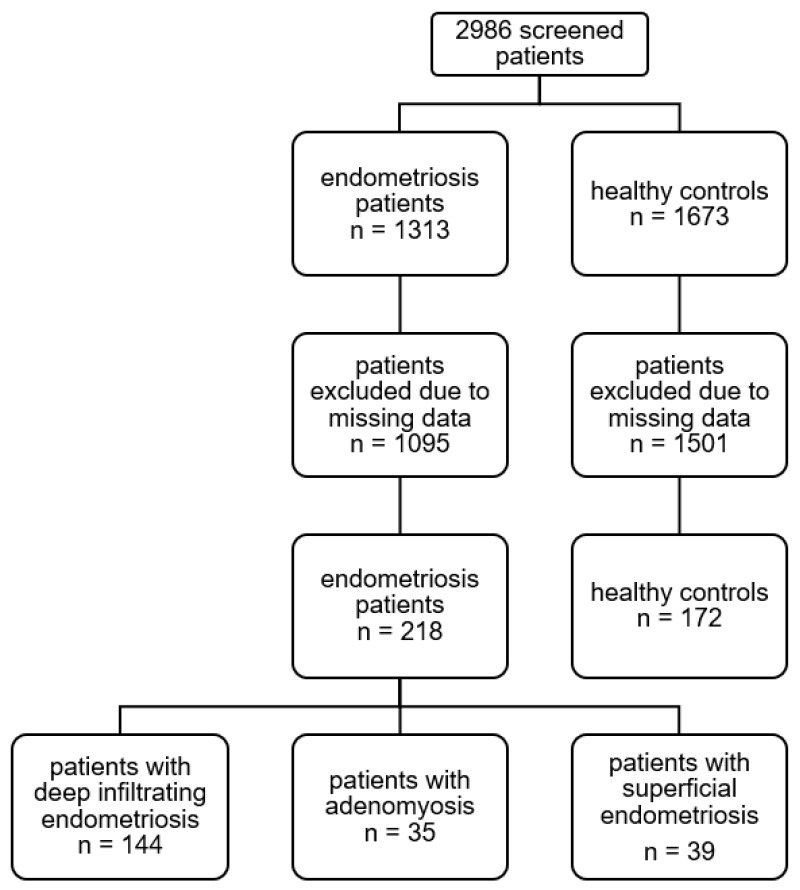
Patient flowchart.

This retrospective, single-center exploratory study was conducted at the Department of Gynaecology, Medical University of Vienna, covering the period from January 2015 to December 2021. The study cohort comprised 390 women of reproductive age who underwent diagnostic or therapeutic laparoscopy due to endometriosis or benign ovarian cysts during this timeframe. Histopathological confirmation was available in all cases. Of these, 218 women were diagnosed with endometriosis, and 172 served as controls with benign ovarian cysts and no evidence of endometriosis (Appendix A.1, Figure A1). To ensure accurate exclusion of endometriosis in the control group, only patients who underwent laparoscopy were eligible. Since surgery is not performed in healthy individuals without indication, patients with simple benign ovarian cysts were considered the most suitable control population. These patients underwent laparoscopy for non-inflammatory, benign conditions, and the absence of endometriosis was confirmed intraoperatively. This approach has also been applied in previous studies evaluating inflammatory or molecular markers in endometriosis research [55,56].

### Study Population

A total of 2986 patients were screened for inclusion in this retrospective study (Appendix A.1, Figure A1). Of these, 1313 patients had a histologically confirmed diagnosis of endometriosis, while 1673 patients served as potential controls. The control group was composed of women who underwent surgical treatment for benign ovarian cysts without evidence of endometriosis upon histological examination. These patients were chosen as controls to ensure that endometriosis could be reliably excluded, as a definitive exclusion of the disease is only possible after laparoscopy and histological assessment.

During the screening process, a substantial number of patients were excluded from both the endometriosis and control groups due to incomplete data, specifically the absence of preoperative laboratory values necessary for calculating the fibrinogen-to-albumin ratio (FAR). In the endometriosis group, 1095 out of 1313 patients (approximately 83.4%) had to be excluded for this reason. Similarly, 1501 out of 1673 control patients (approximately 89.7%) were excluded due to missing laboratory data.

As a result, the final study population included 218 women with histologically confirmed endometriosis and 172 control patients without endometriosis. These patients had complete datasets, including all relevant clinical and laboratory parameters required for the statistical analysis.

Within the cohort of 218 endometriosis patients, subgroup analyses were conducted based on specific disease phenotypes. Among these, 144 patients were diagnosed with deep infiltrating endometriosis (DIE), 35 patients had histologically confirmed adenomyosis, and 39 patients presented with superficial peritoneal endometriosis. These subgroup classifications allowed for exploratory analyses of potential differences in FAR levels according to distinct clinical subtypes of the disease.

This structured screening and selection process ensured that only patients with confirmed diagnoses and complete datasets were included in the final analysis, thereby enhancing the internal validity and reliability of the study findings.

Eligible participants were aged 18 to 50 years and had preoperative laboratory measurements of fibrinogen and albumin available. Exclusion criteria included postmenopausal status, systemic infections such as HIV or hepatitis A, B, or C, chronic liver disease, autoimmune disease, and haematologic disorders. In addition, pregnant and postpartum patients were excluded due to the physiological changes in inflammatory and coagulation parameters during these periods. These criteria were chosen to reduce confounding influences on systemic inflammatory markers. Patients with active malignancies or clinically significant cardiovascular diseases (e.g., acute coronary syndrome, chronic heart failure) were not included in the study population. Although these conditions were not predefined exclusion criteria, they were not present in any of the included cases based on medical record review.

Clinical and demographic data were extracted from electronic medical records, including age, body mass index (BMI), gravidity, parity, and preoperative use of hormonal therapy or analgesics. Laboratory values obtained within one week before surgery comprised fibrinogen (g/L), albumin (g/L), C-reactive protein (CRP, mg/dL), leukocyte and platelet counts, prothrombin time (PT), activated partial thromboplastin time (aPTT), thrombin time (TT), and cancer antigen-125 (CA-125). Surgical and histopathological findings were reviewed to assign disease severity according to the revised American Society for Reproductive Medicine (rASRM) classification and to document the presence of adenomyosis and deep infiltrating endometriosis (DIE). Adenomyosis was analyzed as a separate entity because it is not included in the rASRM classification. Deep infiltrating endometriosis (DIE) is also not adequately captured by rASRM scoring and was therefore addressed as a distinct subgroup.

The fibrinogen-to-albumin ratio (FAR) was calculated for each patient using the formula:

$$FAR=\frac{Fibrinogen\;\left(g/L\right)}{Albumin\;\left(g/L\right)}$$

Statistical analyses were conducted using IBM SPSS Statistics version 27.0 (IBM Corp., Armonk, NY, USA). Normality was assessed with the Kolmogorov–Smirnov test. Continuous variables were summarized as medians with interquartile ranges (IQR) or means with standard deviations (SD), as appropriate. Between-group comparisons were performed using the Mann–Whitney U test for non-normally distributed variables and independent-samples *t*-tests for normally distributed variables. Categorical data was analyzed using the chi-square test. Associations between FAR and rASRM stage were evaluated using Spearman’s rank correlation coefficient (ρ). To evaluate the diagnostic utility of ratio biomarker for endometriosis when compared to ovarian cyst, ROC analysis was performed and the AUC was calculated. To compare FAR across rASRM stages, one-way analysis of variance (ANOVA) was applied with Bonferroni correction for multiple testing. For exploratory analyses of adenomyosis and DIE, one-sided Mann–Whitney U tests were performed, as we hypothesized that FAR would be elevated in these subgroups. Statistical significance was defined as *p* < 0.05 for primary analyses. Exploratory comparisons were interpreted cautiously without adjustment for multiplicity.

## 3. Results

### 3.1. Baseline Characteristics

A total of 390 women met the inclusion criteria. Among them, 218 (55.90%) were diagnosed with histologically confirmed endometriosis, while 172 (44.10%) served as controls with benign ovarian cysts. The median age did not differ significantly between the endometriosis group (32.00 years, IQR 28.00–38.00) and controls (33.50 years, IQR 26.00–41.75; *p* = 0.38). Body mass index was also comparable, with medians of 22.40 kg/m^2^ and 22.60 kg/m^2^, respectively (*p* = 0.37). (Appendix A.2, Table A1).

Preoperative laboratory findings showed significant differences between groups. Women with endometriosis had higher fibrinogen levels (median 2.98 g/L, IQR 2.68–3.44) compared to controls (2.94 g/L, IQR 2.46–3.30; *p* = 0.03). Albumin levels were slightly lower in the endometriosis group (median 44.85 g/L, IQR 42.70–46.73) than in controls (45.20 g/L, IQR 43.30–47.40; *p* = 0.08). C-reactive protein was also elevated in women with endometriosis (median 0.10 mg/dL, IQR 0.04–0.32) compared with controls (0.085 mg/dL, IQR 0.03–0.21; *p* = 0.04). (Appendix A.2, Table A1).

**Figure 1:**
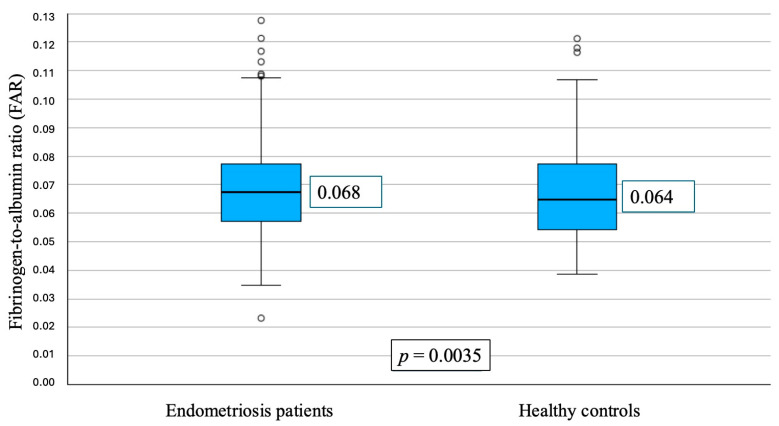
Boxplots comparing the fibrinogen-to-albumin ratio (FAR) between women with endometriosis and control patients. The measure of dispersion is the interquartile range (IQR), and the line inside the box represents the median value of FAR. Group comparisons were performed using the Mann–Whitney U test.

The calculated fibrinogen-to-albumin ratio (FAR) was significantly higher in the endometriosis group (median 0.0679, IQR 0.0588–0.0778) than in controls (0.0641, IQR 0.0559–0.0716; *p* < 0.01, Mann–Whitney U test) (Figure 1).

### 3.2. FAR and Disease Severity

**Figure 2:**
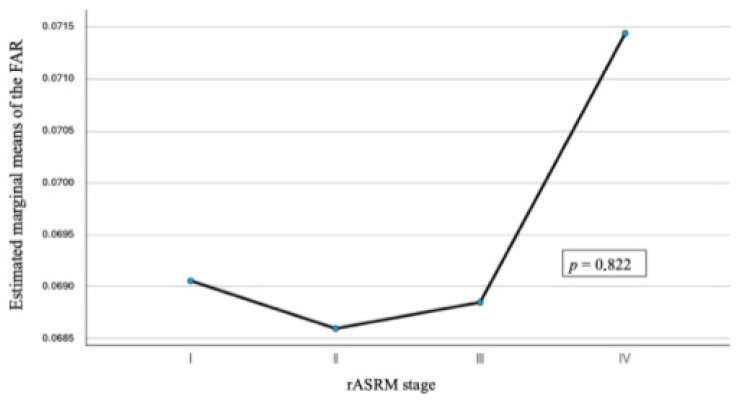
Estimated marginal means of the fibrinogen-to-albumin ratio (FAR) across rASRM stages. The black line connects the estimated marginal means of the fibrinogen–albumin ratio across rASRM stages to illustrate the overall trend. ANOVA demonstrated no statistically significant differences between stages (*p* = 0.82).

To explore whether FAR was associated with disease severity, we examined its distribution across rASRM stages. Of the 218 endometriosis patients, 39 (17.9%) were classified as stage I, 38 (17.4%) as stage II, 54 (24.8%) as stage III, and 74 (33.9%) as stage IV. As shown in Table 1, the mean FAR values were 0.069 ± 0.016 (stage I), 0.069 ± 0.025 (stage II), 0.069 ± 0.018 (stage III), and 0.067 ± 0.018 (stage IV). Median values ranged from 0.065 in stage II to 0.0670 in stage IV, with considerable overlap of interquartile ranges. Minimum and maximum values also indicated wide within-group variability (e.g., 0.035–0.19 in stage II). One-way ANOVA revealed no statistically significant differences in FAR across the four stages (*p* = 0.82). (Figure 2)

**Figure 3:**
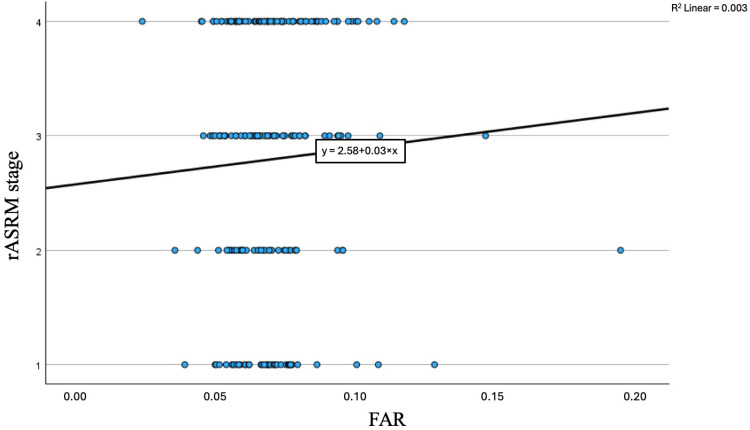
Scatterplot of the fibrinogen-to-albumin ratio (FAR) against rASRM stage. Each dot represents an individual patient. The solid line indicates the fitted linear regression trend (R^2^ = 0.003) calculated using a least-squares model. The regression line is shown for visual reference only. Spearman’s rank correlation revealed no significant association between FAR and rASRM stage (ρ = 0.085, *p* = 0.24).

Consistently, Spearman’s correlation showed no meaningful association between FAR and rASRM stage (ρ = 0.085, *p* = 0.24). A scatterplot of FAR by rASRM stage (Figure 3) confirmed the absence of a clear trend (R^2^ = 0.003).

### 3.3. FAR and Deep Infiltrating Endometriosis

Among the 218 patients, deep infiltrating endometriosis was present in 144 (66.1%), while 39 (17.8%) had only superficial lesions and 35 (16.0%) had confirmed adenomyosis. Median FAR values were 0.068 (IQR 0.059–0.078; range 0.023–0.17) in women with DIE and 0.067 (IQR 0.056–0.077; range 0.045–0.128) in those without DIE. This difference was not statistically significant (*p* = 0.39, one-sided Mann–Whitney U test).

### 3.4. Summary of Findings

Overall, FAR was significantly elevated in women with endometriosis compared to controls. However, no association was observed between FAR and disease severity as measured by rASRM stage, nor with the presence of DIE.

## 4. Discussion

This study explored the fibrinogen-to-albumin ratio (FAR) as a potential inflammatory biomarker in endometriosis by analyzing routinely collected preoperative laboratory data from a well-characterized surgical cohort. We compared FAR values between women with histologically confirmed endometriosis and a control group with benign ovarian cysts who underwent laparoscopy. Our findings showed that FAR was significantly higher in patients with endometriosis, supporting the hypothesis that this ratio reflects systemic inflammatory changes associated with the disease. However, no significant differences in FAR were observed across rASRM stages, suggesting limited utility for disease severity grading. Additionally, subgroup analyses showed no significant association of FAR with deep infiltrating endometriosis (DIE) or adenomyosis.

The observed elevation in FAR supports the established role of systemic inflammation in endometriosis [57]. The condition is characterized by a pro-inflammatory peritoneal environment, involving cytokines such as IL-6 and TNF-α [21], which stimulate hepatic fibrinogen synthesis while suppressing albumin production through altered vascular permeability and reduced protein synthesis [52]. FAR, by integrating these two acute-phase reactants, reflects this imbalance. Similar associations have been described in oncology, cardiovascular, and autoimmune contexts. For example, in coronary artery disease, elevated FAR was associated with increased disease severity and worse prognosis [58]. In various cancers, such as hepatocellular [46], nasopharyngeal [59], and gynecologic malignancies [45,60], high FAR has been associated with poor survival outcomes [61]. In autoimmune diseases like systemic lupus erythematosus, FAR correlates with disease activity [62]. These studies suggest that the ratio between fibrinogen and albumin may capture inflammatory shifts in chronic disease processes more effectively than either marker alone.

This broader literature supports the rationale for investigating FAR in endometriosis, a condition increasingly recognized as involving both local and systemic inflammation [9,11,14].

The pathogenesis and clinical manifestations of endometriosis are influenced by chronic immune activation, increased cytokine production, and impaired immune surveillance [2,20,63]. While CA-125 remains the most widely studied biomarker in this context, its sensitivity and specificity are limited, especially in early-stage disease or in differentiating among phenotypes [35].

The diagnostic performance of FAR in this cohort was modest. The ROC analysis yielded an area under the curve (AUC) of 0.58, indicating limited discriminatory value. While statistically significant, this level of performance is insufficient for clinical application in isolation. Furthermore, the lack of association between FAR and rASRM stage may reflect both the limitations of the rASRM classification—which does not adequately represent phenotypic heterogeneity—and the possibility that systemic inflammation does not scale linearly with lesion burden.

Subgroup analyses of adenomyosis and DIE revealed no statistically significant differences in FAR levels, although a non-significant trend toward higher FAR in patients with adenomyosis was observed. These findings should be interpreted cautiously due to limited subgroup sample sizes and the exploratory nature of the analyses.

This study has several strengths, including a well-characterized cohort, histological confirmation of diagnosis, and preoperative lab data collected under routine clinical conditions.

Limitations include its retrospective, single-center design, lack of clinical symptom data, and absence of multivariable adjustment for confounding factors such as BMI or metabolic status. Subgroup analysis of ovarian and peritoneal endometriosis was not possible, as these subtypes were not systematically classified in our dataset. Specific clinical features such as pain severity, menstrual symptoms, or findings from physical examination were not assessed in this study and therefore could not be analyzed in relation to FAR. Future prospective studies should investigate how FAR correlates with preoperative symptomatology and clinical suspicion. FAR values may also be influenced by subclinical conditions not accounted for in this analysis. In our study, the average age between the groups was not significantly different, but it is known that fibrinogen levels can increase with age [64]. Because of this, age could still be a confounding factor and should be taken into account in future studies with larger numbers of patients.

Importantly, FAR can be derived from standard laboratory tests, and the associated laboratory costs are very low compared to advanced molecular diagnostic tools. This supports the potential of FAR as a cost-effective complementary measure for integration into personalized diagnostic approaches. In our study, we focused on identifying a marker that is easy to use and based on routine laboratory parameters, in contrast to existing advanced molecular diagnostic tools, which are already available but not yet broadly accessible in clinical practice.

Although our results show that FAR is significantly higher in women with endometriosis compared to those without, FAR is unlikely to serve as a standalone marker. Its possible role as an additional marker in a multimodal diagnostic approach is still hypothetical. However, because FAR reflects systemic inflammation, it could be useful in combination with other parameters. Future studies should investigate if FAR improves diagnostic performance when combined with other markers such as CA-125, CRP, IL-6 [13,41], NLR [36,56,65,66], or coagulation factors [36,56,65]. Including FAR in multivariable models could help to find out wether it improves diagnostic accuracy and supports individualized clinical decision-making. Future studies should investigate the stability of FAR over time, its responsiveness to therapy, and its potential to aid in patient stratification, thus advancing personalized management strategies for endometriosis.

## 5. Conclusions

In this retrospective analysis, the fibrinogen-to-albumin ratio was significantly elevated in women with endometriosis compared to controls, consistent with the inflammatory nature of the disease. However, FAR did not correlate with rASRM stage, deep infiltrating endometriosis, or adenomyosis, and its diagnostic accuracy was limited. FAR may nonetheless represent a useful adjunctive parameter within multimodal diagnostic frameworks. Prospective validation and integration into personalized biomarker panels are warranted to clarify its role in individualized diagnosis and management of endometriosis.

## Figures and Tables

**Table 1:** Fibrinogen-to-albumin ratio (FAR) across rASRM stages.

FAR	Mean	SD	Min/Max	*p*-Value
rASRM I	0.069	0.016	0.038/0.123	0.82
rASRM II	0.069	0.024	0.035/0.194	
rASRM III	0.069	0.018	0.045/0.146	
rASRM IV	0.067	0.018	0.023/0.117	

Values are presented as means with standard deviations (SD) and minimum/maximum values. Comparisons between stages were performed using one-way analysis of variance (ANOVA). No statistically significant differences were observed between groups (*p* = 0.82).

## Data Availability

The original contributions presented in this study are included in the article. Further inquiries can be directed to the corresponding author.

## Appendix A

### Appendix A.2. Patient Characteristics

**Table A1:** Patient Characteristics. BMI (Body Mass Index), G (Gravidity), P (Parity), HT (Hormone Therapy), PT (Pain Therapy), rASRM (revised American Society for Reproductive Medicine Score), DIE (Deep Infiltrating Endometriosis), CRP (C-reactive Protein), LEUKO (Leukocytes), THR (Platelets), TT (Thrombin Time), PT (Prothrombin Time), aPTT (Activated Partial Thromboplastin Time), FIB (Fibrinogen), ALB (Albumin), g/L (grams per liter), mg/dL (milligrams per deciliter), sec (seconds), kU/L (kilo-units per liter). * Endometriosis: Percentages (%) refer to the proportion of endometriosis patients among the total study population (*n*). Continuous variables are presented as medians with interquartile ranges (IQR). ** Control: Percentages (%) refer to the proportion of patients with ovarian cysts among the total study population (*n*). Continuous variables are presented as medians with interquartile ranges (IQR). *** Mann–Whitney U test and Chi-square test were applied to compare the distribution between the two patient groups (Endometriosis vs. Control).

	Endometriosis *	Controls **	*p*-Value ***
Number of patients (*n*)	218 (55.90%)	172 (44.10%)	-
Age (median, IQR)	32.00 (28.00–38.00)	33.50 (26.00–41.75)	0.38
BMI (median, IQR)	22.40 (20.60–26.70)	22.60 (20.08–25.05)	0.37
G (median, IQR)	0 (0–1)	0 (0–2)	<0.01
P (median, IQR)	0 (0–1)	0 (0–2)	<0.001
HT (*n*, %)	33 (21.1%)	19 (14.8%)	0.16
PT (*n*, %)	55 (35%)	15 (11.1%)	<0.001
rASRM (*n*, %)			
I	39 (17.9%)	-	-
II	38 (17.4%)	-	-
III	54 (24.8%)	-	-
IV	74 (33.9%)	-	-
Adenomyosis (*n*, %)	35 (16.1%)	-	-
DIE (*n*, %)	144 (66.1%)	-	-
Lab result (median, IQR)			
CRP (mg/dL)	0.10 (0.04–0.32)	0.09 (0.033–0.21)	0.04
LEU (G/L)	6.96 (5.64–8.85)	6.98 (5.63–8.69)	0.74
THR (G/L)	270.50 (238.00–308.00)	263.00 (226.25–302.00)	0.05
TT (s)	16.10 (15.60–16.80)	16.30 (15.70–17.23)	0.16
PT (%)	89.00 (79.75–103.25)	88,50 (77.25–100.75)	0.29
aPTT (s)	34.80 (32.70–37.45)	34.70 (32.40–37.80)	0.89
FIB (g/L)	2.98 (2.68–3.44)	2.94 (2.46–3.30)	0.03
ALB (g/L)	44.85 (42.70–46.73)	45.20 (43.30–47.40)	0.08
CA-125 (kU/L)	36.10 (16.50–89.05)	19.40 (13.30–37.65)	0.01
